# Comparison of emergency cervical cerclage and expectant treatment in cervical insufficiency in singleton pregnancy: A meta-analysis

**DOI:** 10.1371/journal.pone.0278342

**Published:** 2023-02-24

**Authors:** Yanfang Wei, Sumei Wang

**Affiliations:** 1 Department of Obstetrics, Guangxi International Zhuang Medical Hospital, Nanning, Guangxi Zhuang Autonomous Region, China; 2 Department of Obstetrics, The First Affiliated Hospital of Guangxi Medical University, Nanning, Guangxi Zhuang Autonomous Region, China; University of Palermo: Universita degli Studi di Palermo, ITALY

## Abstract

**Objective:**

To compare the therapeutic effects of emergency cervical cerclage and expectant treatment in preterm birth due to cervical insufficiency in singleton pregnancy.

**Methods:**

A combination of subject words and free words was used to search major domestic and foreign databases. According to inclusion and exclusion criteria, 23 studies were included that met the criteria and quality evaluation and data extraction was carried out. The data were analyzed using STATA 15 and the reporting was done in reference to the list of Preferred Reporting Items for Systematic and Meta-Analyses.

**Results:**

Emergency cervical cerclage was superior to expectant treatment for the primary outcome of pregnancy prolongation (WMD = 5.752, 95% CI 5.194–6.311, 22 studies, N = 1435, *I*^2^ = 97.1%, P = 0.000). Cervical cerclage was also superior to expectant treatment for the secondary outcomes of neonatal birth weight (WMD = 1051.542, 95% CI 594.107–1508.977, 9 studies, N = 609, *I*^*2*^ = 96.4%, P = 0.000), neonatal Apgar 1′ (WMD = 2.8720, 95% CI: 2.105–3.639, 11 studies, N = 716, *I*^*2*^ = 99.0%, P = 0.000), number of live births (OR = 6.018, 95% CI 2.882–12.568, 10 studies, N = 724, *I*^*2*^ = 55.3%, P = 0.000), deliveries after 32 weeks (OR = 8.030, 95% CI 1.38–46.892, 8 studies, N = 381, *I*^*2*^ = 85.9%, P = 0.021). deliveries after 34 weeks (OR = 15.91, 95% CI 5.92–42.77, 9 studies, N = 560, *I*^*2*^ = 59.6%, P = 0.000), number of vaginal deliveries (OR = 3.24, 95% CI 1.32–7.90, 8 studies, N = 502, *I*^*2*^ = 69.4%, P = 0.018), and number of neonatal survivals (OR = 9.300, 95% CI 3.472–24.910, 10 studies, N = 654, *I*^*2*^ = 80.5%, P = 0.000). No difference between emergency cervical cerclage and expectant treatment was found in patients with chorioamnionitis (OR = 1.85, 95% CI 0.602–4.583, 4 studies, N = 296, *I*^*2*^ = 16.3%, P = 0.273).

**Conclusion:**

Before the 28th week of pregnancy, emergency cervical cerclage can significantly prolong the gestational week and improve the neonatal survival rate, compared to expectant treatment, in women with singleton pregnancies who have a dilated uterine orifice caused by cervical insufficiency.

## 1. Introduction

Late miscarriage and preterm birth (PTB) are one of the most common perinatal complications. It is estimated that 1,168,126 babies are born prematurely each year in China, which ranks second in the world [1]. Notably, preterm neonatal mortality accounts for approximately three-quarters of perinatal mortality [2], and late miscarriage and PTB have become the leading causes of neonatal death. Among the causes of miscarriage and premature delivery in late pregnancy, cervical insufficiency (CI) is one of the most important. The incidence of CI in pregnant women ranges from 0.1% to 2.0%, while it is as high as 15% in patients with recurrent miscarriages in the second trimester and 8%─9% in patients with late miscarriages and PTB [3].

Surgery and conservative treatment are the two main types of treatment for CI. Cervical cerclage (CC), currently the sole effective surgical treatment of CI, includes prophylactic CC and emergency CC (ECC). Prophylactic CC is performed upon indication in medical history and ultrasonography and is usually performed between 12 and 14 weeks of gestation [4]. In contrast, ECC is usually performed after the cervix has dilated [5]. Conservative treatment of CI includes expectant treatment, progesterone, and pessary treatment. Previous studies reported that progesterone reduces the risk of PTB by maintaining the cervical length. Therefore, the use of vaginal progesterone can effectively prevent PTB [6, 7].

Since the advent of CC, the safety and efficacy of preventive CC has been widely recognized [8] and it is recommended by the latest guidelines [4]. However, whether to choose ECC or conservative treatment for those with a dilated cervix is still under debate.

Supporters of ECC believe that its’ safety and effectiveness have been greatly improved in recent years with adjuvant therapy and perioperative medication. In addition, some studies indicate that ECC and prophylactic CC have similar effects when antibiotics and tocolytics are used properly [9–13]. However, the effectiveness of conservative treatment, in comparison to ECC, is less reported and lacks sufficient evidence-based medical evidence.

Proponents of conservative treatment believe that its success rate has been greatly improved in recent years via the application of tocolytics. However, there is no unified conclusion about the safety and efficacy of conservative treatment due to the difficulty in determining the optimal timing and complex procedures. Emerging evidence indicates that ECC, compared with prophylactic CC, has poor pregnancy outcomes and significantly increases complication rates [14–17]. Furthermore, the effectiveness of perioperative medication has also been questioned by several studies due to the insufficient evidence of tocolytics usage after ECC [18, 19] and failure in controlling infection with antibiotics after cerclage [20].

Currently, there are no available guidelines regarding whether to choose ECC or conservative treatment for patients with dilated cervix due to CI. A search of the Cochrane Library showed that are only five registered randomized controlled trials (RCTs) that compared the two treatments since 2000, of which two RCTs are still under investigation [21–25]. Therefore, the lack of standardized guidelines and sufficient evidence-based medical evidence forces physicians to compel diagnosis and treatment plans based on personal experience, which not only leads to inconsistent procedures but also excessive treatment or treatment delay.

Thus, the current meta-analysis was performed to compare the reliability and effectiveness between ECC and expectant treatment in CI-mediated preterm birth, which could provide evidence for the treatment of such patients and promote the formulation of medical decisions.

## 2. Materials and methods

The Preferred Reporting Items for Systematic Reviews and Meta-analysis (PRISMA) statement was used in this study with adjustments based on the characteristics of meta-analysis.

### 2.1. Inclusion and exclusion criteria

RCTs or observational studies published after 2005 were included.

### 2.2. Participants and interventions

#### 2.2.1. Participants were patients with singleton pregnancies and asymptomatic (no labor pain, no contractions, and no significant vaginal bleeding) dilation of the cervix and/or shortening of the cervix length

Patients with multiple pregnancies and/or with premature rupture of membranes or clinical symptoms of chorioamnionitis were excluded.

#### 2.2.2. Interventions include ECC or conservative treatment

ECC could be performed by McDonald’s procedure or Shirodkar’s procedure, while bed rest was considered as conservative treatment. Usage of the following adjuvant drugs alone or in combination was included: antibiotics, tocolytics, and progesterone.

### 2.3. Outcomes

#### 2.3.1. Primary outcome measurement

Prolonged pregnancy duration.

#### 2.3.2. Secondary outcome measurements

Neonatal birth weight, neonatal 1-minute Apgar score, number of live births, number of deliveries >32 weeks, number of deliveries >34 weeks, number of vaginal deliveries, number of surviving neonates at 28 days, and number of patients with chorioamnionitis.

### 2.4. Search strategy

Four English-language databases (PubMed, EMBASE, The Cochrane Library, and Web of Science) and four Chinese-language databases (China Biomedical Literature Database (CBM), China National Knowledge Infrastructure (CNKI), WanFang Data, and Chongqing VIP) were searched for studies from 2005 to present. English-language databases were searched for relevant articles using the following words or phrases: ((Cervical Insufficiency [mh]) OR (Cervical Incompetence, Uterine) OR (Incompetence, Uterine Cervical) OR (Incompetent OR (Cervices, Incompetent) OR (Cervix, Incompetent) OR (Incompetent Cervices) OR (Cervix Cervix) Incompetence) OR (Incompetence, Cervix)) AND ((Emergency Cervical Cercla-ge[mh]) OR (Cerclage of Uterine Cervix) OR (Uterine Cervix Cerclage) OR (Cervical Cerclage) OR (Cerclage of Cervix))) AND ((expectant management [mh]) OR (Waiting, Watchful) OR (Expectant Management) OR (Management, Expectant) OR (Active Surveillance) OR (Surveillance, Active)). Chinese-language databases were searched for relevant articles using the following words or phrases: (keyword: cervical insufficiency) AND (keyword: emergency cervical cerclage). In addition, the search scope was expanded by carefully reading the references of the retrieved studies, and eligible studies were retrieved as comprehensively as possible. All studies were carefully compared to avoid the inclusion of duplicate or overlapping articles. In the case of overlap, studies with the largest number of cases were included.

### 2.5. Assessment of risk of bias

Literature screening, data extraction, and quality evaluation were completed independently by two researchers. In case of discrepancies in the search results, the two researchers resolved the disagreement by consulting a third party.

#### 2.5.1. Selection of studies

For each study, preliminary relevance was determined by screening the title and abstract. Further inclusion and exclusion criteria were formulated according to the PICOS principles: (1) Outcome indicators included prolonged pregnancy, gestational age at delivery, number of live births, number of spontaneous deliveries, neonatal 1-minute Apgar score, 28-day survival of neonates, birth weight of neonates, chorioamnionitis and other index values; (2) Exclusion of non-original data, incomplete original data, or original data not in the form of standard deviation. According to the above inclusion and exclusion criteria, the full text of the selected studies was read, and studies that finally met the criteria were selected.

#### 2.5.2. Data extraction

Extracted data were incorporated into a database including the following information: baseline demographics, basic characteristics and exclusion criteria of the study, the applied intervention, and its effectiveness.

#### 2.5.3. Quality evaluation of study

The quality of the included observational studies was evaluated using the Newcastle–Ottawa scale (NOS) with a maximum score of 10. Studies with a score of at least 5 or more were included in this study. Cochrane risk of bias analysis was performed to evaluate the quality of RCTs. Studies were divided into low risk of bias, high risk of bias, and uncertain risk of bias groups. Studies with a high risk of bias represented extremely low reference value.

### 2.6. Statistical analysis

#### 2.6.1. Stata15 software was used to perform the meta-analysis, develop a forest plot, and to perform a heterogeneity test on the included studies

The heterogeneity between the included studies was analyzed by the chi-squared (χ2) test (test level α = 0.1) and Higgins *I*^*2*^ statistics. If there was no statistical heterogeneity between the results of each study (P>0.1 and I^2^<50%), the fixed-effect model was used for meta-analysis. If there was statistical heterogeneity (P≤0.1, I^2^>50%), the source of heterogeneity was further analyzed, and the random-effect model was used for analysis.

#### 2.6.2. Pooled sample statistics

For numerical variables, the weighted mean difference (WMD) was used for the pooled statistic. For binary variables, the odds ratio (OR) was used for the pooled statistic. To test whether the pooled statistic was statistically significant, we performed the following analysis: (1) Confidence interval method: firstly, a 95% confidence interval (95% CI) was calculated. When the effect index was WMD, there was no statistical significance if the 95% CI contained 0; there was statistical significance if the upper and lower limits of the 95% CI did not contain 0. When the effect index was OR, there was no statistical significance if the 95% CI contained 1; there was statistical significance if the upper and lower limits of the 95% CI did not contain 1; (2) The z-test: the probability (P) value of the statistic was obtained according to the z value. If P≤0.05, the pooled statistic of multiple studies was statistically significant. In contrast, if P>0.05, the pooled statistic of multiple studies had no statistical significance.

#### 2.6.3. Sensitivity analysis and subgroup analysis

Sensitivity analysis was performed on the results of the meta-analysis to evaluate the stability and reliability of the results. Subgroup analysis was performed on the results of the meta-analysis to determine the source of heterogeneity, and to study whether a variable had an impact on the results.

#### 2.6.4. Analysis of publication bias

Begg’s and Egger’s tests were performed to test for publication bias.

## 3. Results

### 3.1. Search results

**Fig 1:**
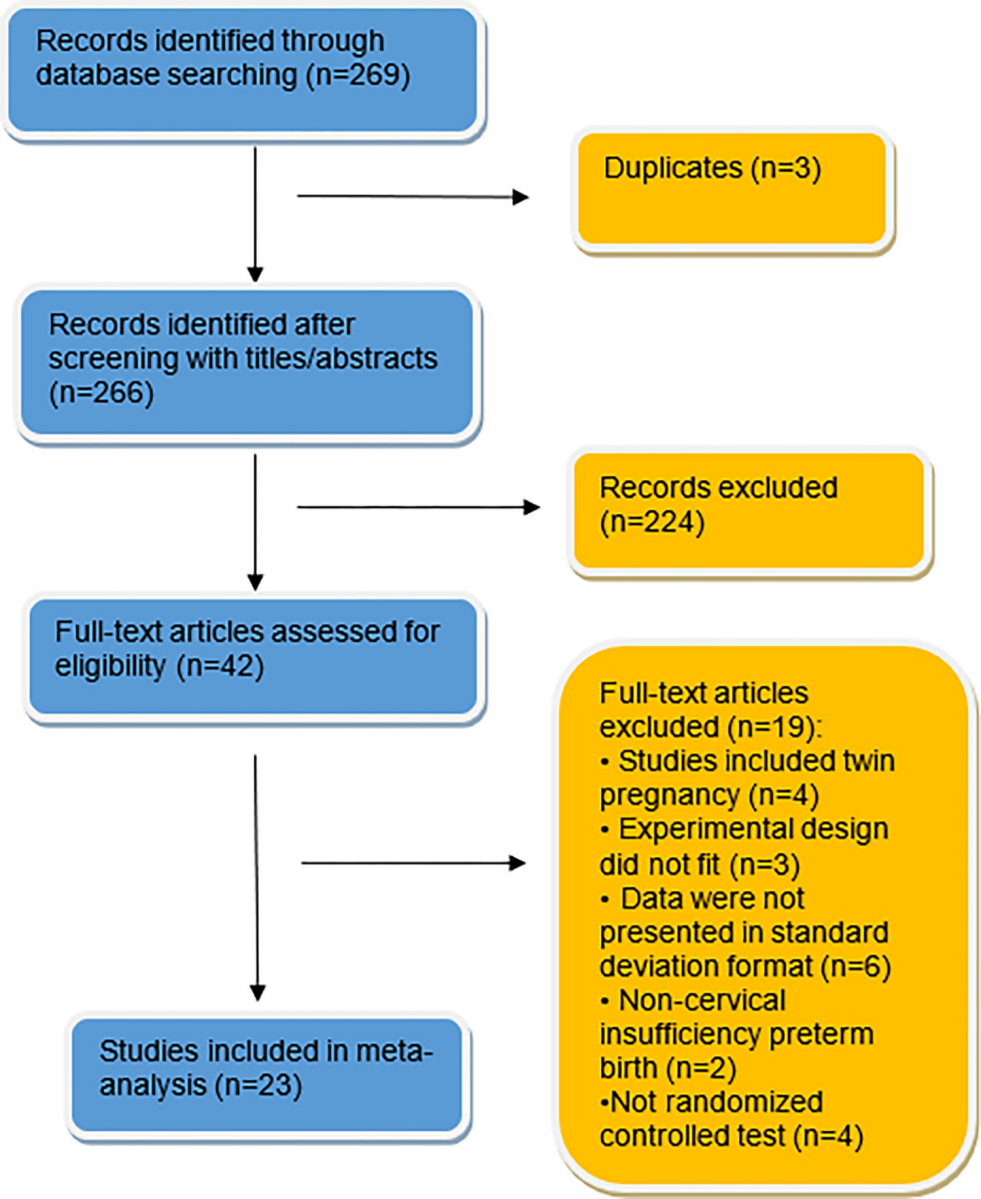
Flow chart of the selection procedure.

A total of 269 potential studies were retrieved through the established literature retrieval strategy, of which 42 studies were obtained after a preliminary screening of the title and abstract. Two researchers read the obtained studies individually and checked them carefully according to the inclusion and exclusion criteria. After screening, 23 studies were included in this meta-analysis [22, 23, 26–46], of which 18 studies were from Chinese databases and 5 from English databases. The screening process is shown in Fig 1.

### 3.2. Literature quality and general characteristics

The methodological quality of studies was assessed with the NOS and Cochrane risk of bias assessment tool. General characteristics of the data retrieved from the 23 studies are as follows: For the ECC used in the observation group, 12 studies reported the McDonald’s technique, 2 studies reported the Shirodkar’s technique, and 1 study reported both techniques. The other 8 studies did not explicitly mention the procedures used for ECC. Except for the primary treatment methods (ECC in the observation group, while bed rest in the control group), 13 studies used antibiotics to prevent infection. Five of the studies reported antibiotic usage in both observation and control groups, while 8 studies reported the use of antibiotics in the observation group only. In 18 studies, patients of both the observation and control groups were treated with tocolytics including magnesium sulfate, ritodrine, and salbutamol. Progesterone was given to patients via intravenous drip, intramuscular injection, oral administration, and vaginal administration in 5 studies. The general characteristics of the included studies and medication management are shown in Table 1.

**Table 1:** Characteristics of the included studies.

Author, year	Age (year)	No. of Pregnancy	No. of Delivery	Gestational at inclusion	Cervical Condition	No. of Cases		Antibiotics	Tocolytics	Progesterone	Total Score
						Cerclage Group	Conservative Group				
Liu et al 2016 [26]	21–38	2±1.28	1.5±0.28	N/A	N/A	50	20	N/A	N/A	N/A	Low risk
Guo et al, 2021	21–39	N/A	N/A	16–30 wk	Length<3 cm, dilation >2 cm	42	42	Used	Used	N/A	Low risk
Zhou et al, 2016 [34]	25–38	N/A	N/A	16–25 wk	Length<2.5 cm, dilation <3 cm	53	57	Used	Used	Used	6
Cui et al, 2012	21–39	6-Feb	2-Jan	N/A	Length≤2.5 cm, dilation <1 cm	40	25	N/A	Used	N/A	6
Sha et al, 2016 [30]	22–38	4-Jan	N/A	14–31 wk	N/A	15	15	N/A	Used	N/A	Low risk
Zhang et al, 2018 [31]	30.3±4.4	1.84±0.97	0.29±0.49	13–28 wk	Length<2 cm	47	21	Used	Used	N/A	8
Liu et al 2019 [32]	21–40	N/A	N/A	N/A	N/A	59	58	Used	Used	N/A	6
Liu et al 2019 [33, 36]	23–38	N/A	N/A	22–31 wk	Enlargement 2–5 cm	18	16	Used	Used	N/A	6
Zhao et al, 2014 [34]	31.8±3.5	2.43±1.28	0.24±0.43	12–28 wk	Length<1.5 cm, dilation <3 cm	36	39	Used	Used	N/A	8
Liao et al, 2019 [35]	22–38	5-Feb	0–2	17–30 wk	Dilation <3 cm	39	39	Used	Used	N/A	Low risk
Liu et al, 2019 [33, 36]	20–38	N/A	N/A	17–26 wk	N/A	31	31	N/A	Used	N/A	Low risk
Pei et al, 2020	20–38	N/A	N/A	17–26 wk	Length<2.5 cm, dilation ≥1.5 cm	40	40	Used	Used	N/A	7
Han et al, 2019 [38]	23–36	N/A	N/A	15–31 wk	Length<2.5 cm	20	21	N/A	Used	Used	Low risk
Ragab et al, 2015 [23]	30.9±3.1	1.82±0.94	0.26±0.48	24–28 wk	Length increase ≥50%, dilation <5 cm	50	50	Used	N/A	Used	Low risk
Ciavattini et al, 2015 [39]	34.2±4.5	N/A	N/A	14–24 wk	Dilation ≥1 cm	18	19	Used	Used	Used	8
Daskalakis et al, 2006	26.8±4.1	N/A	N/A	18–26 wk	Length<1.5 cm	29	17	Used	N/A	N/A	7
Costa et al, 2019 [40]	26.9±7.1	N/A	N/A	16–28 wk	Dilation 1–3 cm	19	11	N/A	N/A	N/A	6
Aoki et al, 2013 [41]	27–42	N/A	N/A	15–26 wk	Dilation 1–4 cm	15	20	Used	Used	N/A	6
Zuo et al, 2017 [42]	22–38	3.62±1.86	N/A	22.1±5.9 wk	N/A	30	18	N/A	Used	N/A	7
Li et al, 2016 [46]	20–39	4-Jan	N/A	25.3±0.24 wk	Dilation 2–4 cm	32	31	N/A	Used	N/A	7
Liang et al, 2017	20–39	6-Jan	0–3	15–27 wk	N/A	40	40	Used	Used	N/A	Low risk
Mai et al, 2014	22–39	3±2	N/A	22–31 wk	N/A	38	37	N/A	Used	N/A	Low risk
Wang et al, 2020 [45]	22–38	N/A	N/A	18–24 wk	N/A	28	28	Used	N/A	Used	Low risk

### 3.3. Meta-analysis results

#### 3.3.1. Prolonged pregnancy

**Fig 2:**
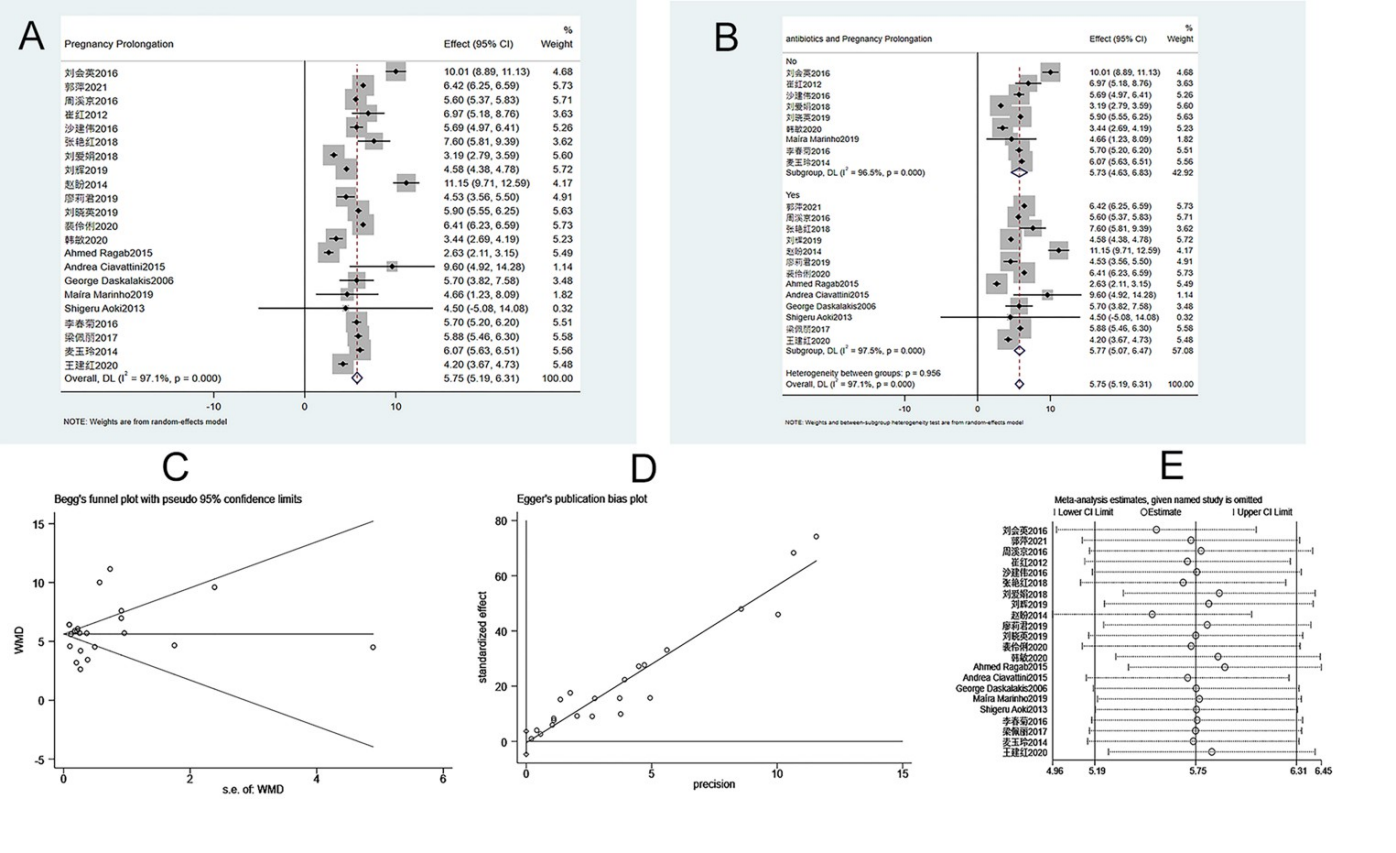
Meta-analysis of pregnancy prolongation. A. Forest Plot. B. Subgroup analysis of antibiotics. C. Begg’s test. D. Egger’s test. E. Sensitivity analysis.

A total of 22 case-controlled studies were included in the analysis. The heterogeneity test (I^2^ = 97.1%, P = 0.000) showed that there was heterogeneity among studies. Therefore, a random-effects model was used. As shown in Fig 2A, the random-effects meta-analysis showed that ECC is significantly better than conservative treatment in prolonging pregnancy duration (WMD = 5.752, 95% CI 5.194–6.311, P = 0.000). Subgroup analysis suggested that the use of antibiotics had no effects on the prolongation of pregnancy (Fig 2B). Similarly, subgroup analysis showed that the use of tocolytics had no significant effects on the prolongation of pregnancy (Fig 2C). Sensitivity analysis suggests that the meta-analysis results are stable and reliable (Fig 2F). The Begg’s test (P = 0.955) and Egger’s test (P = 0.801) indicated that publication bias was low (Fig 2D and 2E).

#### 3.3.2. Neonatal birth weight

**Fig 3:**
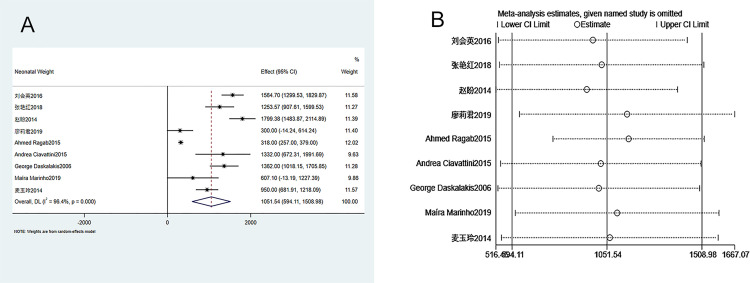
Meta-analysis of neonatal birth weight. A. Forest Plot. B. Sensitivity analysis.

Nine studies were included in the analysis of neonatal birth weight between groups. The heterogeneity test (I^2^ = 96.4%, P = 0.000) showed that there was heterogeneity among studies. Therefore, a random-effects model was used. Fig 3A shows that the neonatal birth weight is significantly higher in the ECC group in comparison to the conservative treatment group (WMD = 1051.542, 95% CI 594.107–1508.977, P = 0.000). Sensitivity analysis suggests that the meta-analysis results are stable and reliable (Fig 3B). Since the number of included studies was less than 10, publication bias analysis was not performed.

#### 3.3.3. Neonatal 1-minute Apgar score

**Fig 4:**
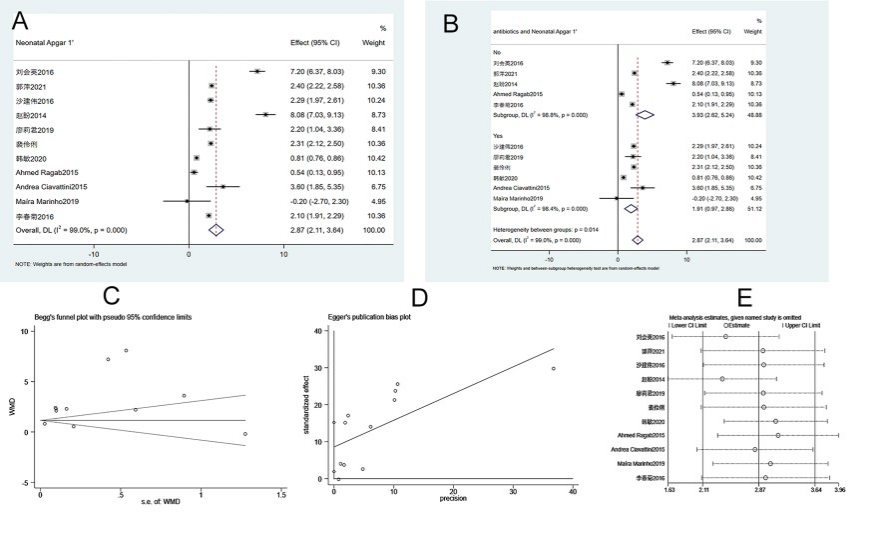
Meta-analysis of the 1-minute Apgar score in neonates. A. Forest Plot. B. Subgroup analysis of antibiotics. C. Begg’s test. D. Egger’s test. E. Sensitivity analysis.

Eleven studies were included in the analysis of neonatal 1-minute Apgar score. The heterogeneity test (I^2^ = 99%, P = 0.000) showed that there was heterogeneity among studies. Therefore, a random-effects model was applied. As shown in Fig 4A, the 1-minute Apgar score of newborns in the ECC groups is significantly higher than that in conservative treatment groups (WMD = 2.8720, 95% CI 2.105–3.639, P = 0.000). Subgroup analysis suggested that the use of antibiotics had no effects on the neonatal 1-minute Apgar score between groups (Fig 4B). Begg’s test (P = 0.533) and Egger’s test (P = 0.017) indicated that publication bias was found in the studies (Fig 4C and 4D). Sensitivity analysis suggests that the meta-analysis results are stable and reliable (Fig 4E).

#### 3.3.4. Number of live births

**Fig 5:**
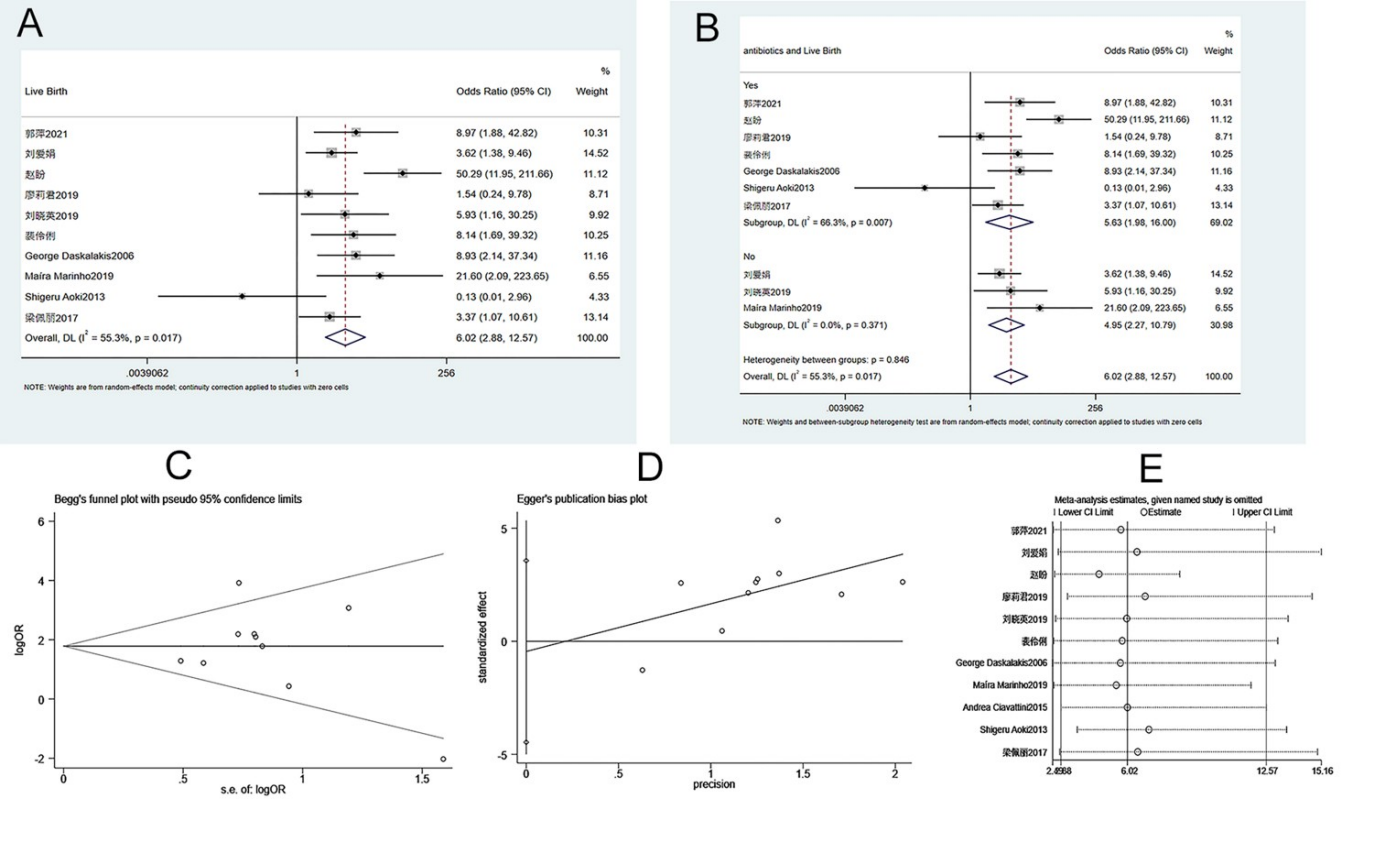
Meta-analysis of number of live births. A. Forest Plot. B. Subgroup analysis of antibiotics. C. Begg’s test. D. Egger’s test. E. Sensitivity analysis.

A total of 10 case-controlled studies were included in the analysis of the number of live births between groups. The heterogeneity test (I^2^ = 55.3%, P = 0.017) showed that there was heterogeneity among studies. Therefore, a random-effects model was applied. As shown in Fig 5A, the number of live births in patients who received ECC is significantly higher than that in the conservative treatment group (OR = 6.018, 95% CI 2.882–12.568, P = 0.000). Subgroup analysis suggested that the use of antibiotics did not affect the number of live births between groups (Fig 5B). The Begg’s test (P = 0.592) and Egger’s test (P = 0.802) showed that the publication bias is low (Fig 5C and 5D). Sensitivity analysis suggests that the meta-analysis results are stable and reliable (Fig 5E).

#### 3.3.5. Number of deliveries (>32 weeks of gestation)

**Fig 6:**
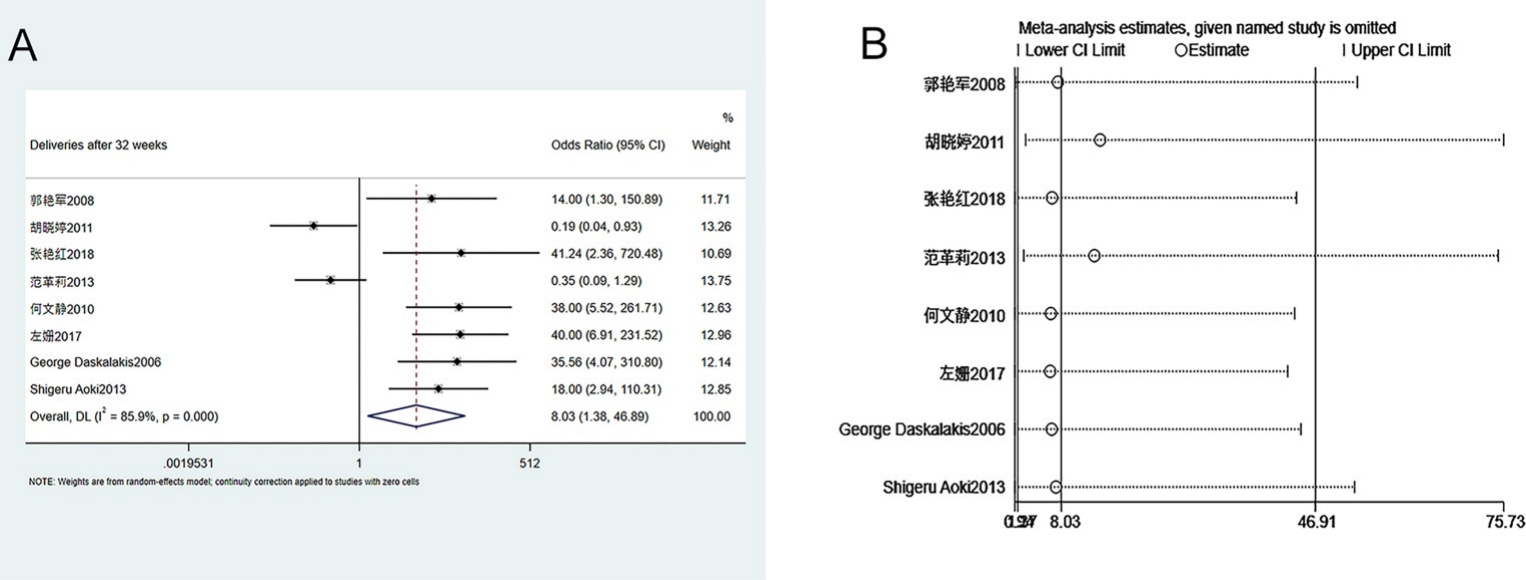
Meta-analysis of deliveries greater than 32 weeks of gestation. A. Forest Plot. B. Sensitivity analysis.

Eight studies were enrolled in the analysis of number of deliveries s (>32 weeks of gestation) between groups. The heterogeneity test (I^2^ = 85.9%, P = 0.000) showed that there was heterogeneity among studies. Therefore, a random-effects model was used. As shown in Fig 6A, the number of deliveries (>32 weeks of gestation) in patients who received ECC is significantly higher than that of the conservative treatment group (OR = 8.030, 95% CI 1.38–46.892, P = 0.021). Sensitivity analysis suggests that the meta-analysis results are stable and reliable (Fig 6B). Since the number of enrolled studies was less than 10, publication bias analysis was not performed.

#### 3.3.6. Number of deliveries (>34 weeks of gestation)

**Fig 7:**
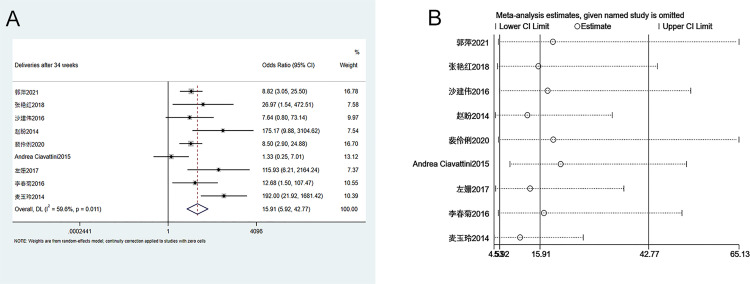
Meta-analysis of deliveries greater than 34 weeks of gestation. A. Forest Plot. B. Sensitivity analysis.

Nine studies were included in the analysis of the number of deliveries (>34 weeks of gestation) between groups. The heterogeneity test (I^2^ = 59.6%, P = 0.011) showed that there was heterogeneity among studies. Therefore, a random-effects model was used. As shown in Fig 7A, the number of deliveries (>34 weeks of gestation) in patients who received ECC is significantly higher than that of the conservative treatment group (OR = 15.91, 95% CI 5.92–42.77, P = 0.000). Sensitivity analysis suggests that the meta-analysis results are stable and reliable (Fig 7B). Since the number of enrolled studies was less than 10, publication bias analysis was not performed.

#### 3.3.7. Number of vaginal deliveries

**Fig 8:**
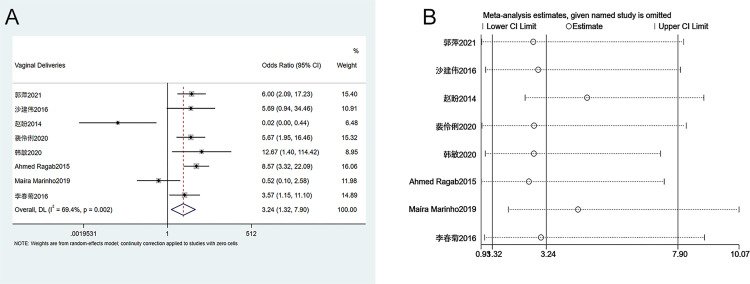
Meta-analysis of the number of vaginal deliveries. A. Forest Plot. B. Sensitivity analysis.

Eight studies were included in the analysis of the number of vaginal deliveries. The heterogeneity test (I^2^ = 69.4%, P = 0.002) showed that there was heterogeneity among studies, and a random effects model was used. As Fig 8A indicates, the number of vaginal deliveries in patients who received ECC is significantly higher than that of the conservative treatment group (OR = 3.24, 95% CI 1.32–7.90, P = 0.018). Sensitivity analysis suggests that the meta-analysis results are stable and reliable (Fig 8B). Since the number of enrolled studies was less than 10, publication bias analysis was not performed.

#### 3.3.8. Number of neonatal 28-day survivors

**Fig 9:**
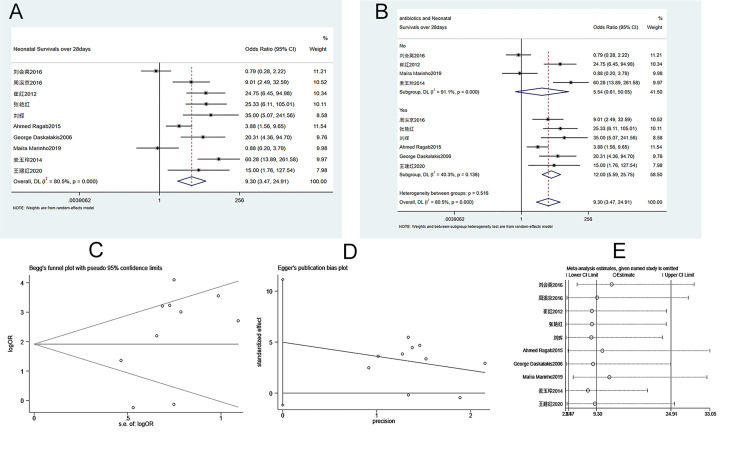
Meta-analysis of neonatal 28-day survival. A. Forest Plot. B. Subgroup analysis of antibiotics. C. Begg’s test. D. Egger’s test. E. Sensitivity analysis.

A total of 10 case-controlled studies were included in the analysis of the number of newborns who survived the first 28 days. The heterogeneity test (I^2^ = 80.5%, P = 0.000) showed that there was heterogeneity among studies, and a random effects model was used. As shown in Fig 9A, a significantly higher number of neonatal 28-day survivors was found in the ECC group compared to the conservative treatment group (OR = 9.300, 95% CI 3.472–24.910, P = 0.000). Subgroup analysis suggested that the use of antibiotics increased the number of neonatal 28-day survivors when compared to patients who did not receive antibiotics (Fig 9B). The Begg’s test (P = 0.371) and Egger’s test (P = 0.099) showed that the publication bias is low (Fig 9C and 9D). Sensitivity analysis suggests that the meta-analysis results are stable and reliable (Fig 9E).

#### 3.3.9. The incidence of chorioamnionitis

**Fig 10:**
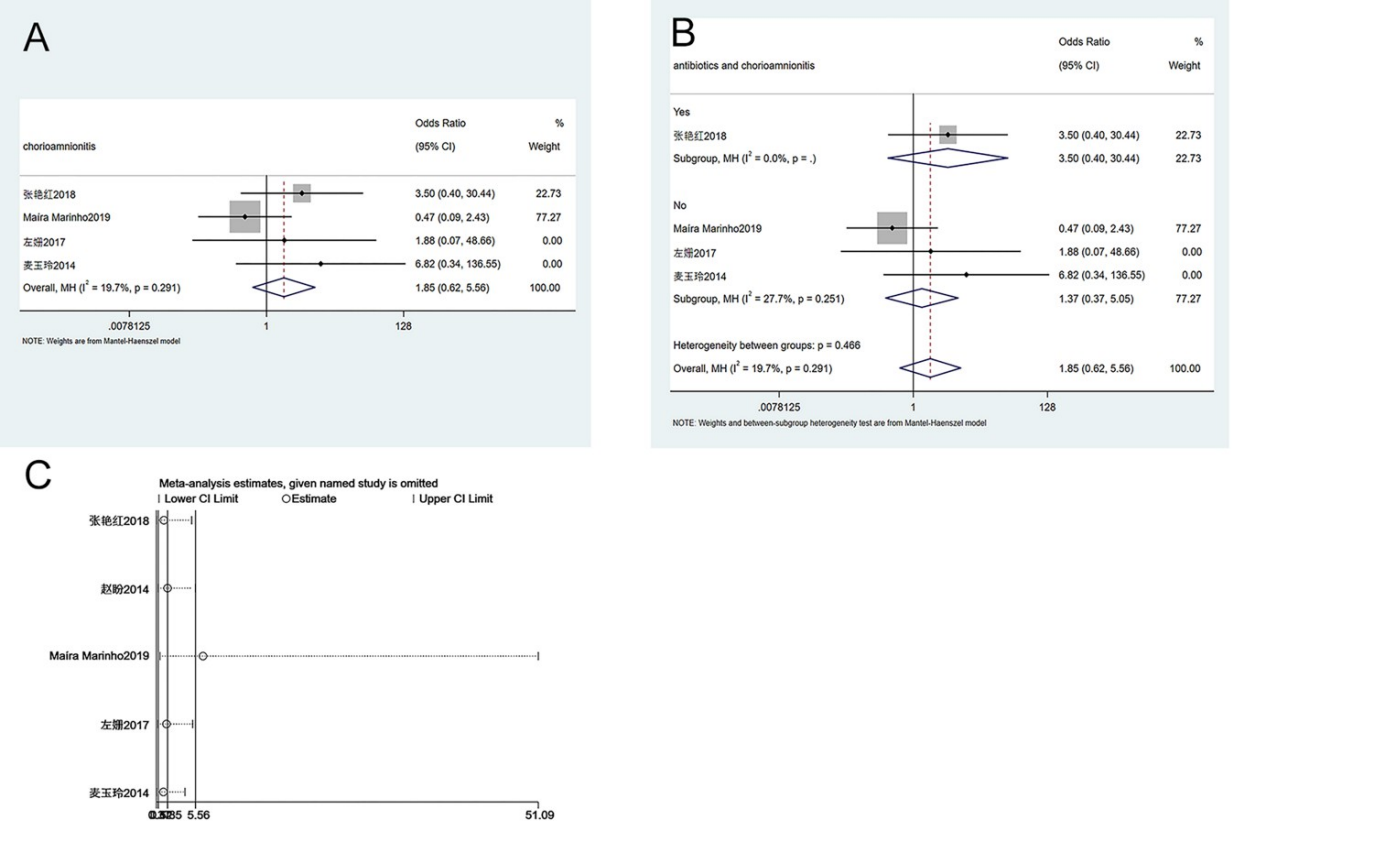
Meta-analysis of chorioamnionitis incidence. A. Forest Plot. B. Subgroup analysis of antibiotics. C. Sensitivity analysis.

A total of 4 case-controlled studies were included in the analysis of chorioamnionitis incidence between groups. The heterogeneity test (I^2^ = 19.7%, P = 0.291) showed that there was no heterogeneity among studies, and a fixed-effect model was applied. As shown in Fig 10A, there is no significant difference in chorioamnionitis incidence between patients receiving ECC and patients receiving conservative treatment (OR = 1.85, 95% CI 0.62–5.56, P = 0.273). Subgroup analysis suggested that the use of antibiotics had no effects on chorioamnionitis incidence between groups (Fig 10B). Sensitivity analysis suggests that the meta-analysis results are stable and reliable (Fig 10C). Since the number of enrolled studies was less than 10, publication bias analysis was not performed.

## 4. Discussion

Surgery and conservative treatment are common options for pregnant women at risk for late miscarriage or PTB due to CI. Surgical treatment included ECC, while conservative treatment include expectant treatment, progesterone treatment, and pessary treatment. Of the 23 studies included in our meta-analysis, ECC was classified as the observation group, and expectant treatment was classified as the control group. Patients in 4 control studies received progesterone treatment [23, 31, 39, 45].

The pessary technique was initially developed as early as 1977 [47]. After the advent of the pessary technique, various kinds of pessaries with different materials, sizes, and shapes have been used to prevent PTB [48–54]. Previous studies reported that the pessary is effective in managing CI [55, 56]. However, the number of relevant studies is still too small to allow a systematic analysis. Therefore, studies related to pessaries were excluded from the current analysis. In addition, the etiology and mechanism of miscarriage/PTB in twin pregnancies is complicated and require different treatment procedures. To reduce the influence of complicated factors on the analysis, we also excluded studies containing twin pregnancies.

### 4.1. Main findings

Our meta-analysis shows that, compared with conservative treatment, ECC can significantly prolong pregnancy duration and significantly reduce the risk of PTB at <32 weeks, <34 weeks, and <37 weeks, which is consistent with the 2019 The Society of Obstetricians and Gynaecologists of Canada (SOGC) guidelines [4].

In terms of neonatal outcomes, ECC has obvious advantages over conservative treatment in improving live birth rate, neonatal birth weight, neonatal 1-minute Apgar score, and neonatal 28-day survival rate. The 2021 American College of Obstetricians and Gynecologists (ACOG) Preterm Birth Prevention Guidelines mentioned that ECC had "potential neonatal benefit" [2]. Notably, our meta-analysis concluded that ECC is of great benefit to neonates. During our study screening, only 6 studies related to maternal complications were obtained, of which 5 studies reported the chorioamnionitis condition [31, 34, 40, 42, 44]. Our analysis showed that there was no significant difference in the incidence of chorioamnionitis between the two groups. Unfortunately, other complications, such as premature rupture of membranes and cervical tear, could not be statistically analyzed because the obtained data was too limited. Similarly, due to the limited sample size (only 2 studies) of neonatal complications, statistical analysis was not performed. Interestingly, we found that ECC increased the number of vaginal deliveries when compared to conservative treatment. In addition, we explored the effects of tocolytics and antibiotics on patient outcomes. Our data suggested that the use of tocolytics had no significant effects on outcomes in patients receiving either ECC or conservative management. Although the use of antibiotics had no effects on prolonged pregnancy, the number of live births, neonatal 1-minute Apgar score, and the 28-day survival of neonates were improved with antibiotics treatment.

### 4.2. Analysis and comparison

The results of our study indicate that ECC significantly prolongs the gestational age in comparison to conservative treatment. This conclusion is supported by many previous studies [57–59], while some other studies reached different conclusions. For example, Alfirevic’s study indicated that ECC could only reduce the rate of PTB, while it did not affect perinatal mortality and neonatal morbidity [60]. However, our meta-analysis concluded that ECC was superior to conservative management in terms of prolonged gestation and neonatal 28-day survival. Our conclusion is evidenced by improved gestational age, birth weight, and Apgar score of the newborn, which are closely related to the long-term survival of the newborn. Although statistical analysis was not performed due to insufficient data, two included studies by the Costa and Ciavatini groups suggested that neonatal morbidity was significantly lower following ECC in comparison to conservative treatment. However, further RCTs with large samples should be performed to confirm this observation.

Çavuş et al. previously reported that there was no difference in delivery method between ECC and conservative treatment [61]. In contrast, our meta-analysis concluded that the rate of vaginal delivery after ECC was higher than that of conservative treatment. One possible explanation is that ECC effectively prolongs the gestational age, which increases the tolerance of birth canal compression, thereby reducing the probability of transferring to cesarean section during vaginal trial labor due to fetal distress. In addition, we hypothesize that the level of local medical care could also result in differences. More rigorously designed randomized controlled experiments are needed to further confirm the effects of ECC on delivery methods.

Previous studies have suggested that ECC itself increases the risk of infection [62–64]. In contrast, our meta-analysis indicates that ECC and conservative treatment showed no significant differences in the incidence of chorioamnionitis. It is possible that an infection was caused by a pre-existing lesion rather than the surgery. Thus, chorioamnionitis after cerclage may not be entirely attributed to the procedure itself. Due to the limited number of included studies, our conclusions should be validated by future studies. Since there was no significant difference in infection rates between the two groups, the use of antibiotics becomes controversial. To address this critical question, we performed a subgroup analysis and concluded that antibiotic usage did not affect outcomes in terms of prolonged pregnancy, the number of live births, neonatal 1-minute Apgar score, and chorioamnionitis incidence. However, antibiotics showed beneficial effects on the 28-day survival of newborns when compared to the group without antibiotics. We hypothesize that since cerclage itself does not increase the risk of infection, the use of antibiotics after ECC and conservative treatment does not affect the incidence of chorioamnionitis. Of note, substantial evidence indicates that approximately 68% of CI is associated with intrauterine infection, and the incidence of infection rises to 80% in acute CI cases [65–68]. Therefore, regardless of ECC or conservative treatment, rational use of antibiotics is recommended because preventive treatment of latent infections that may exist can reduce neonatal morbidity and improve long-term neonatal survival. This statement is consistent with a previous study by Bayrak et al. [69]. Nonetheless, successful detection and diagnosis of potential infections with the assistance of advanced testing techniques could provide accurate use of antibiotics. However, considering the difficulty of identifying pre-existing infection, we recommend using broad-spectrum antibiotics to reduce the incidence of infections and further improve neonatal outcomes.

The use of tocolytics is also under debate. Theoretically, painless cervical dilation caused by CI is attributed to a functional or structural defect of the cervix, which is essentially different from preterm labor caused by uterine contractions. Given that there are no uterine contractions in CI, it is worth investigating whether tocolytics are necessary as treatment. The 2019 SOGC Guidelines for CI state that indomethacin may be used before cervical cerclage in order to suppress the dilatation of fetal membranes by reducing the amount of amniotic fluid and suppressing uterine contractions, which may increase the success rate of cervical cerclage [21]. Except for indomethacin, no other tocolytics were suggested in the Guidelines. Interestingly, tocolytics as adjuvant drugs are often used in both ECC and conservative treatment during clinical diagnosis and treatment. For ECC, tocolytics are prophylactically used because the patient’s uterus is in an irritable state after cerclage. CI leads to dilation of the cervix and subsequent mechanical pulling of the cervix, which causes reflex contractions. Therefore, tocolytics are also used in conservative treatment. Currently, there is no direct evidence to support the use of tocolytics in these patients. We found that tocolytics, including ritodrine hydrochloride, magnesium sulfate, Tosiban, and albuterol were used in most of the included studies (18 out of 23). We performed subgroup analysis to assess whether tocolytics had any impact on prolonging pregnancy. The results showed that the use of tocolytics did not affect pregnancy prolongation. This conclusion provides a basis for future investigations.

The 2021 ACOG Guidelines for Spontaneous PTB recommends ECC at 16–24 weeks gestational age [3]. Interestingly, we found that ECC was applied to patients at more than 24 weeks of gestational age in 15 included studies, and the maximum gestational age even reached 31 weeks. However, sensitivity analysis indicated that larger gestational age had no impact on the effectiveness of ECC. In contrast, ECC performed at late gestational age was associated with higher neonatal weight, Apgar score, and neonatal survival. Importantly, a recent study by Cao et al. reported that the neonatal survival rates at the gestational ages of <25 weeks, 26–27 weeks, 28–29 weeks, and 30–31 weeks are 65.6%, 89%, 94.9%, and 98.3%, while the rates of serious complication are 89.5%, 73.2%, 48.9%, and 30.7%, respectively [70]. These findings suggest that the probability of serious complications remains at high levels (73.2%─89.5%). In addition to CI, fetal growth restriction is also an important cause of PTBs as it accounts for approximately 30% of all PTBs and is the most common pregnancy complication [71, 72]. Although the neonatal survival rate of PTB that occurs between 24 and 28 weeks of gestation is greater than 50%, it could cause a huge economic burden to the family and society. Therefore, based on our meta-analysis, we believe that if the gestational age is between 24 and 28 weeks, ECC can still be considered after thorough communication with the patient and their informed consent in order to maximize the improvement of neonatal outcomes.

### 4.3. Strengths and limitations

To ensure the inclusion of high-quality studies and obtain convincing conclusions, we searched as many eligible studies as possible and carried out a strict quality evaluation strategy. However, there are still some limitations that may affect our conclusions. First, not all the included studies were RCTs. Thirteen studies were observational studies [22, 28, 29, 31–34, 38–43], which may introduce bias and cause distorted results. In addition, six included RCTs did not clearly describe the specific random allocation method [26, 30, 36, 38, 44, 45], which may also introduce bias. Second, since the application of cervical cerclage in multiple pregnancy is controversial and only a few cervical cerclage cases in multiple pregnancies have been reported, we only included studies related to singleton pregnancies. Hence, some well-designed high-quality studies could have been missed during the search. Third, due to the lack of objective and unified international standards for the diagnosis of CI, the measurements, such as cervical canal length, degree of cervix dilation, and gestational age are not unified. A more rigorous experimental design is needed to further verify whether the differences in these measurements could cause high heterogeneity in our study. Fourth, due to the limited information on maternal complications and long-term prognosis of neonates, we could only focus on outcome indicators in systematic analysis, such as prolongation of gestational age, neonatal birth weight, and Apgar score, while maternal and neonatal outcomes could not be accounted for. As mentioned in the 2021 ACOG guidelines, if ECC turns miscarriage into premature delivery, cerclage will not only be less beneficial but also increase the medical costs and risks to patients. Therefore, it is expected that more studies will focus on the maternal and neonatal outcomes of patients who receive ECC or conservative treatment to provide a basis for the formulation of optimal medical strategies. Fifth, among the 23 included studies, 18 studies reported the use of tocolytics. However, the types of tocolytics used across the studies were different. Whether different types, usages, and doses of tocolytics will affect the conclusions should be evaluated in future studies. Sixth, although we searched as many English-language databases as possible, most of the included studies (18 studies) were carried out in China, and only 5 studies were retrieved from English-language databases. One possible explanation is that the concept of PTB treatment is different in China. Fetal lung maturation treatment within 48 hours, rather than an extension of gestational age by drugs or surgery is commonly adopted in other countries, which results in fewer cases of cervical cerclage use in comparison to China. Therefore, it is relevant that location bias could impact the quality of studies and increase the study bias, which subsequently reduces the reliability of our study. Seventh, due to incomplete information, it is difficult to specifically analyze and discuss other factors, such as age, race, underlying diseases, and nutritional status in the current study.

## 5. Conclusions

Based on our meta-analysis, the following conclusions can be drawn: (1) For women with singleton pregnancy who have a dilated cervix due to CI, ECC can significantly prolong the gestational age and improve the neonatal survival rate before 24 weeks of gestation when compared to conservative treatment.

(2) Sensitivity analysis suggests that ECC at a later gestational age (between 24 and 28 weeks) could have beneficial effects and improve neonatal outcomes. However, data on the efficacy of ECC in women at later gestational age is very limited, and more studies are needed to reach a reliable conclusion. Therefore, clinicians should be cautious, but ECC in this population can still be considered to improve neonatal outcomes.

(3) ECC does not increase the incidence of chorioamnionitis.

(4) ECC does not increase the rate of cesarean section.

(5) The use of tocolytics is not necessary.

(6) The use of antibiotics does not improve prolongation of the gestational age but can improve the 28-day survival rate of neonates.

## Supporting information

S1 Checklist PRISMA 2020 checklist. (DOCX)
